# Exploring the circulating metabolome of sepsis: metabolomic and lipidomic profiles sampled in the ambulance

**DOI:** 10.1007/s11306-024-02172-5

**Published:** 2024-10-05

**Authors:** Samira Salihovic, Daniel Eklund, Robert Kruse, Ulrika Wallgren, Tuulia Hyötyläinen, Eva Särndahl, Lisa Kurland

**Affiliations:** 1 School of Medical Sciences, Faculty of Medicine and Health, Örebro University, Örebro, Sweden; 2 Inflammatory Response and Infection Susceptibility Centre (iRiSC), Örebro University, 701 82 Örebro, Sweden; 3 Department of Clinical Research Laboratory, Faculty of Medicine and Health, Örebro University, Örebro, Sweden; 4 Department of Clinical Science and Education, Karolinska Institutet, Stockholm, Sweden; 5 School of Science and Technology, Örebro University, Örebro, Sweden

**Keywords:** Sepsis, Infection, Ambulance, Metabolomics, Lipidomics, Plasma

## Abstract

**Background:**

Sepsis is defined as a dysfunctional host response to infection. The diverse clinical presentations of sepsis pose diagnostic challenges and there is a demand for enhanced diagnostic markers for sepsis as well as an understanding of the underlying pathological mechanisms involved in sepsis. From this perspective, metabolomics has emerged as a potentially valuable tool for aiding in the early identification of sepsis that could highlight key metabolic pathways and underlying pathological mechanisms.

**Objective:**

The aim of this investigation is to explore the early metabolomic and lipidomic profiles in a prospective cohort where plasma samples (n = 138) were obtained during ambulance transport among patients with infection according to clinical judgement who subsequently developed sepsis, patients who developed non-septic infection, and symptomatic controls without an infection.

**Methods:**

Multiplatform metabolomics and lipidomics were performed using UHPLC–MS/MS and UHPLC–QTOFMS. Uni- and multivariable analysis were used to identify metabolite profiles in sepsis vs symptomatic control and sepsis vs non-septic infection.

**Results:**

Univariable analysis disclosed that out of the 457 annotated metabolites measured across three different platforms, 23 polar, 27 semipolar metabolites and 133 molecular lipids exhibited significant differences between patients who developed sepsis and symptomatic controls following correction for multiple testing. Furthermore, 84 metabolites remained significantly different between sepsis and symptomatic controls following adjustment for age, sex, and Charlson comorbidity score. Notably, no significant differences were identified in metabolites levels when comparing patients with sepsis and non-septic infection in univariable and multivariable analyses.

**Conclusion:**

Overall, we found that the metabolome, including the lipidome, was decreased in patients experiencing infection and sepsis, with no significant differences between the two conditions. This finding indicates that the observed metabolic profiles are shared between both infection and sepsis, rather than being exclusive to sepsis alone.

**Supplementary Information:**

The online version contains supplementary material available at 10.1007/s11306-024-02172-5.

## Introduction

Sepsis is manifested through a dysregulated host response to infection, leading to life-threatening organ dysfunction (Singer et al., 2016). Early recognition and intervention are crucial for treatment and improved outcomes. In a study from 2020, the authors reported that there were 48.9 million sepsis patients worldwide, resulting in 11 million deaths and accounting for 19.7% of the total death toll (Rudd et al., 2020). The World Health Organization identifies sepsis as a global health priority and urges countries to implement strategies in order to reduce its global burden (Reinhart et al., 2017).

The standard treatment for sepsis is early recognition and prompt administration of broad-spectrum antibiotics and supportive care, including fluid resuscitation (Singer et al., 2016). However, although there have been many advances in treatment strategies for sepsis in recent years, the mortality rate remains high, and may be explained, in part, by the pathophysiology involved in sepsis progression remaining ambiguous. There is a need for improved and more precise molecular characterization of sepsis that can aid in early diagnosis, predictions of prognosis and ensuring that patients can receive timely and effective interventions.

Metabolomics enables analysis of metabolites in circulation and the relationship between metabolites and physiological and pathological changes. Most of the analytes are small molecules with molecular weights of less than 1500 Da that can be used as indicators of physiological or pathological states and to better understand the occurrence and progression of diseases (Wishart, 2019). Previous studies have identified both increased and decreased levels of certain metabolites (phenylalanine, tyrosine, tryptophan, and arginine, alongside with decreased levels of various lysophosphatidylcholines) in the context of sepsis, septic shock, and mortality (Chen et al., 2022; Cho et al., 2012; Mickiewicz et al., 2014; Reisinger et al., 2021). However, one notable challenge lies in the use of diverse metabolomics methodologies, such as proton nuclear magnetic resonance (1H-NMR), gas chromatography–mass spectrometry (GC–MS), and liquid chromatography–mass spectrometry (LC–MS), across these studies. While these techniques offer distinct advantages, variations in sensitivity and metabolite coverage can introduce discrepancies in the detected metabolites. Additionally, many previous studies were characterized by relatively small sample sizes and primarily relied on blood samples collected upon arrival at the emergency department, and not early samples obtained during ambulance transit. Notably, to our knowledge, there is a paucity of research investigating metabolomics and lipidomics profiles using blood samples collected in ambulances and comparing them to symptomatic controls also obtained during ambulance transit. This represents an important gap in the current understanding of metabolic changes during the early stages of sepsis. Therefore, investigating metabolic profiles from the first available contact with sepsis, i.e. during the pre-hospital phase, and using multiple metabolomics platforms to broaden coverage, provides a metabolic snapshot that could improve early identification, which remains challenging with clinical variables alone and may lead to additional insights of pathological metabolic profiles.

Thus, the aim of this study is to explore the early metabolomic and lipidomic profiles in plasma samples from patients with sepsis compared to symptomatic controls and patients with non-septic infections, using plasma samples collected in the ambulance.

## Methods

### Study population

**Fig. 1:**
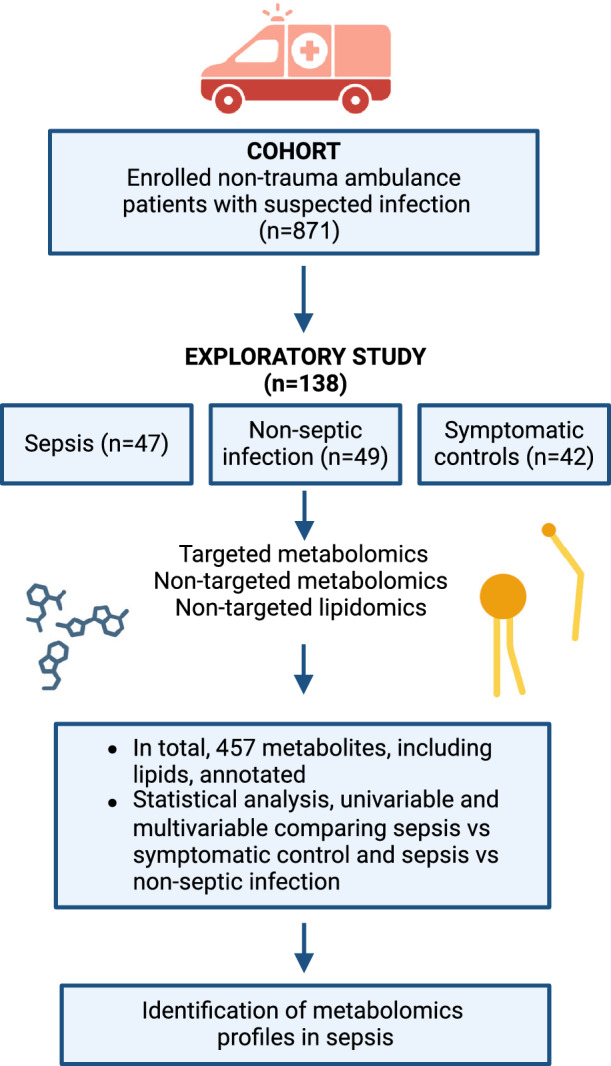
Outline of the study design and analytical workflow of non-trauma ambulance patients with suspected infection. Created with Biorender

This current exploratory investigation is an ancillary study of the larger Predict Sepsis study, a prospective cohort study in the ambulance setting, with patient inclusion between 2017 and 2018, in Stockholm County, Sweden (Wallgren et al., 2020). The study received approval from the Stockholm Regional Ethical Review Board (reference number 2016/2001-31/2, 2018/2202, and 2024-02746-02). Written informed consent was obtained from all participants. The study was registered at ClinicalTrials.gov, identifier: NCT03249597. An overview of the study design is illustrated in Fig. 1. All ambulances were staffed with at least one nurse specialist and one emergency medical technician. Ambulance personnel were blinded to the results of the blood tests.

### Inclusion criteria

Inclusion criteria were adult (≥ 18 years) non-trauma patients with and without infection according to clinical judgment by ambulance personnel. For details see Wallgren et al. (36).

### Exclusion criteria

Exclusion criteria were: (1) lack of written consent; (2) trauma other than fall at home; (3) patient leaving ED prior to physician assessment; (4) direct admission to geriatric hospital i.e., bypassing the ED; (5) missing hospital records, (6) missing personal identification number and (7) missing categorization with respect to infection or not upon inclusion.

### Outcome categories

Participants with and without infection were included in the ambulance. The outcome categories sepsis, non-septic infection, and symptomatic controls. Sepsis was defined in accordance with the Sepsis-3 criteria (Singer et al., 2016), within 36 h from ED arrival including criteria for infection, for details see Wallgren et al. (2020). Infection was defined in accordance with prior criteria (Wallgren et al., 2020). The symptomatic controls are patients that do not fulfill the above criteria for sepsis and infection.

### Demographic variables

Age, sex, and data required for the calculation of Charlson comorbidity score (Charlson et al., 1994) were extracted from hospital records as previously described (Wallgren et al., 2020, 2022).

### Vital signs

The initial measurements of six vital signs, respiratory rate, oxygen saturation, heart rate, systolic blood pressure, Glasgow coma scale (GCS), and temperature, were collected in the ambulance and are provided in Table 1.

**Table 1:** Baseline demographic and clinical characteristics of study population

	Infection		Symptomatic control	*P*-value
	Sepsis	Infection		
N	49	47	42	
Age (median, IQR)	80 (73–87)	77 (68–83)	74 (63–82)	0.1020
Sex (%, female)	40	36	61	0.036
Ambulance vital parameters (median, IQR)				
Respiratory rate (min^−1^)	24 (18–30)	20 (16–22)	16 (16–18)	0.0001
Oxygen saturation (%)	94 (90–96)	96 (93–97)	97 (94–98)	0.0001
Heart rate (min^−1^)	96 (80–110)	88 (78–103)	80 (72–91)	0.0051
Systolic BP (mmHg)	124 (110–150)	130 (115–148)	148 (127–160)	0.0182
GCS (score)	15 (15–15)	15 (15–15)	15 (15–15)	0.2439
Temperature (°C)	38.5 (37.4–39.1)	37.8 (37.4–38.7)	36.7 (36.3–37.1)	0.0001
Blood tests (median, IQR)				
P-Glucose (mmol/L)	7.6 (6.8–8.8)	7.3 (6.4–8.8)	6.9 (6.3–8.2)	0.1672
P-Lactate (mmol/L)	1.7 (1.4–2.3)	1.5 (1.2–2.1)	1.5 (1.1–2.1)	0.0823
P-suPAR (ng/mL)	5.1 (2.8–6.4)	4.2 (2.8–6.4)	2.7 (2.2–3.4)	0.0001
P-HBP (ng/mL)	12.8 (5.9–39.5)	10.5 (5.9–16.6)	5.9 (5.9–9.7)	0.0006
Comorbidity				
Charlson score, median (IQR)	2 (1–4)	2 (1–4)	1 (0–3)	0.0447

*IQR* inter quartile range, *P-* plasma, *suPAR* soluble urokinase Plasminogen Activator Receptor, *HBP* Heparin-Binding Protein, *GCS* Glasgow coma scale

### Blood tests

All blood samples were drawn in the ambulance. Four blood tests were included: plasma-glucose, plasma-lactate, plasma-heparin-binding protein (P-HBP), and plasma-soluble urokinase plasminogen activator receptor (P-suPAR). Glucose was included due to its use in a prior sepsis screening tool for ambulances, lactate for its established role as a sepsis and mortality marker frequently used in Swedish emergency departments, and HBP and suPAR for their promising potential as sepsis biomarkers at the study’s outset (Linder et al., 2015; Robson et al., 2009). The blood samples were tested for these analytes in certified hospital and university laboratories upon arrival at the emergency department and are provided in Table 1. Detailed procedures for handling and analysis are available in the original study by Wallgren et al. (2020).

### Sample collection for metabolomics

Blood was drawn in EDTA-tubes in the ambulance. Upon arrival to the hospital bound emergency departments (EDs), tubes were centrifuged, aliquoted, and plasma was frozen in − 70 °C in biobank.

### Targeted UHPLC–MS/MS metabolomics

Briefly, 100 µL plasma for each individual sample was added to an Ostro 96-well plate well followed by internal standards (D3-lactic acid, D4-3-hydroxybutyric acid, D3-serine, D4-succinic acid, D5-glutamine, D7-arginine, D5-tryptophan) in 450 µL acetonitrile. The filtrate was evaporated to dryness and reconstituted in 50 µL methanol and water (1:1) followed by the addition of recovery standards. Ultra-high pressure liquid chromatography-tandem mass spectrometry measurements were performed on a Waters Acquity UHPLC TQS MSMS. 2 µL of the sample extract was injected onto a 1.7 µm, 2.1 mm × 150 mm Acquity BEH AMIDE HILIC column, in combination with a VAN GUARD-FIT guard column. The column temperature was 30 °C. The gradient mobile phases were, A (20 mM ammonium formate in 100% Milli-Q and 0.1% formic acid) and B (0.1% formic acid in 100% acetonitrile). The flow rate was 0.3 mL/min and the total run time per sample was 21 min. The analysis was performed using electrospray ionization, operating in the positive ion mode. The source temperature was 150 °C and the desolvation temperature was 350 °C. The cone gas flow was 150 L/h and the desolvation gas flow was 650 L/h. The multiple reaction monitoring (MRM) acquisition mode was selected for the relative quantification of the metabolites with an individual span time of 0.1 s, given in their individual MRM channels.

### Non-targeted UHPLC–Q-TOF-MS metabolomics

Sample preparation was performed in the same manner as for the targeted metabolomics described above. The instrumental analysis was performed with UHPLC–Q-TOF-MS (Agilent Technologies, Santa Clara, CA, The United States of America) with an Acquity UPLC, BEH C18 (2.1 × 100 mm, 1.7 μm) (Waters, Milford, MA, USA) column set at 50 °C with a C18 precolumn (Waters, Wexford, Ireland). The mobile phases are as follows: (A) 2 mM NH4Ac in H2O/MeOH (70:30, v/v) and (B) 2 mM NH4Ac in MeOH. The samples were kept at 10 °C, and injection volume was 10 μL. The flow rate was 0.4 mL/min, and the gradient started with 95% A and 5% B with a change after 1.5 min to 70% A and 30% B, which followed a change after 4.5 min to 30% A and 70% B; the last change was after 7.5 min with 100% B until the end of run. Dual-jet stream electrospray (dual ESI) ion source was used and with the ion polarity set on the negative mode. The capillary voltage and the nozzle voltage were kept at 4500 and 1500 V, respectively. The N2 pressure was set at 21 psi, with the sheath gas flow at 11 L/min and temperature at 379 °C for the nebulizer.

### Non-targeted UHPLC–Q-TOF-MS lipidomics

#### Sample preparation and analysis

Plasma samples (10 µL) were extracted with a modified version of the previously published Folch procedure (Nygren et al., 2011). In short, 10 µL of 0.9% NaCl and, 120 µL of CHCl_3_:MeOH (2:1, v/v) containing the internal standards (c = 2.5 µg/mL) was added to each sample. The standard solution contained the following compounds: 1,2-diheptadecanoyl-sn-glycero-3-phosphoethanolamine (PE(17:0/17:0)), *N*-heptadecanoyl-D-erythro-sphingosylphosphorylcholine (SM(d18:1/17:0)), *N*-heptadecanoyl-D-erythro-sphingosine (Cer(d18:1/17:0)), 1,2-diheptadecanoyl-sn-glycero-3-phosphocholine (PC(17:0/17:0)), 1-heptadecanoyl-2-hydroxy-sn-glycero-3-phosphocholine (LPC(17:0)) and 1-palmitoyl-d31-2-oleoyl-sn-glycero-3-phosphocholine (PC(16:0/d31/18:1)), were purchased from Avanti Polar Lipids, Inc. (Alabaster, AL, USA), and, triheptadecanoylglycerol (TG(17:0/17:0/17:0)) was purchased from Larodan AB (Solna, Sweden). The samples were vortex mixed and incubated on ice for 30 min after which they were centrifuged (9400×*g*, 3 min). 60 µL from the lower layer of each sample was then transferred to a glass vial with an insert and 60 µL of CHCl_3_:MeOH (2:1, v/v) was added to each sample. Instrumental analyses were carried out on an ultra-high-performance liquid chromatography quadrupole time-of-flight mass spectrometry method (UHPLC–Q-TOF-MS from Agilent Technologies (Santa Clara, CA, USA) as described previously. The analysis was done on an ACQUITY UPLC® BEH C18 column (2.1 mm × 100 mm, particle size 1.7 µm) by Waters (Milford, USA). Mobile phases were as follows A: 10 mM NH4Ac and 0.1% formic acid in water and B: 10 mM NH4Ac and 0.1% formic acid in acetonitrile/isopropanol (1:1). Dual jet stream electrospray (dual ESI) ion source was used and with the ion polarity was set on positive mode. Internal standard mixture was used for normalization and lipid-class specific calibration (0.1–10 µg/mL) was used for quantitation as previously described. MS data processing was performed using open-source software MZmine 2.52 (Pluskal et al., 2010).

### Lipidomics and non-targeted metabolomics data processing

UHPLC–UHPLC–Q-TOF-MS data were processed independently the targeted UHPLC–MS/MS metabolomics data. Mass spectrometry data was pre-processed with MZmine 2.53 (Pluskal et al., 2010). Peak detection with a noise level of 1000 was performed first, following with ADAP chromatogram builder including group intensity threshold to be as noise level (1000), minimum highest intensity 10 and m/z tolerance 0.009 m/z or 8 ppm. Next, chromatogram deconvolution was performed with local minimum search as algorithm with a 70% chromatographic threshold, 0.05 min of minimum retention time range, 5% minimum relative height, 1000 minimum absolute height, 1,05 as minimum ratio of peak top/edge and peak duration range in minutes from 0.08 to 5.00. Isotopic peak grouper was done next with m/z tolerance of 0.05 m/z or 5 ppm, tR tolerance was set on 0.05 min and a maximum charge of 2. For the alignment of peak lists, a Join alignment algorithm was performed with m/z tolerance as 0.006 or 7 ppm and a weight of m/z as 2 with a retention time tolerance of 0.15 and a weight of 1. Filtering with feature list rows filter was done next with three steps. First step rows that match with all criteria were kept with a retention time range from 0.5 to 12 and m/z of 100–1200. Second filtering step removed rows that match with all criteria with a tR range of 2–4 and m/z of 800–1200. Third filtering step removed rows that match with criteria with a tR range of 4–8 and m/z of 370–500. Next, gap filling with peak finder was done with m/z tolerance of 0.006 m/z or 8 ppm, tR tolerance of 0.17 min and with intensity tolerance as 50%. Identification was done based on in-house library (mz, MS/MS, retention times). Normalization was done by using internal standards: lipids: PE(17:0/17:0), SM(d18:1/17:0), Cer(d18:1/17:0), LPC(17:0), TG(17:0/17:0/17:0) and PC(16:0/d30/18:1)) for identified lipids and closest ISTD for the unknown lipids followed by calculation of the concentrations based on lipid-class concentration curves. For polar and semipolar metabolites, the following ISTDs were used valine-d8, glutamic acid-d5, succinic acid-d4, heptadecanoic acid, lactic acid-d3, citric acid-d4. 3-hydroxybutyric acid-d4, arginine-d7, tryptophan-d5, glutamine-d5, 1-D4-CA, 1-D4-CDCA, 1-D4-CDCA, 1-D4-GCA, 1-D4-GCDCA, 1-D4-GLCA, 1-D4-GUDCA, 1-D4-LCA, 1-D4-TCA, 1-D4-UDCA. For data filtering, we have removed compounds that were present at blank samples (peak area < 5 times that of blank) and compounds that had RSD > 30% in the pooled quality control samples. An overlap exists between the metabolomics and lipidomics methodologies, particularly in the analysis of semi-polar lipids such as lysophosphatidylcholines (LPCs). Isomers of molecular lipids are annotated with and asterisk (*).

### Statistical analysis

The demographic and clinical characteristics are presented as numbers (percentages) for categorical variables and the median with interquartile range (IQR) for continuous variables. The chi-squared test was used to assess differences between binary variables. The Kruskal–Wallis test was used to compare the three study groups for each of the measured variables. The metabolomics and lipidomics analysis yielded 457 metabolites and molecular lipids that could be matched to known annotations. These 457 metabolites and molecular lipids were retained for subsequent statistical analysis. First, the metabolomic and lipidomics variables were log-transformed and mean centred and SD-scaled. Second, Wilcoxon rank‐sum test and false discovery rate (FDR) correction *P* < 0.05 was performed to assess the metabolomic and lipidomic differences between sepsis vs symptomatic control and sepsis vs non-septic infection. Third, multivariable logistic regression with adjustment for age, sex, and Charlson comorbidity score and FDR-correction (*P* < 0.05) was performed for sepsis vs symptomatic control and sepsis vs infection. ***Statistical analyses were performed in Stata 16.1 (StataCorp LLC, TX, USA). Lastly, pathway analysis was performed using MetaboAnalyst 6.0 (Pang et al., 2024). The annotated metabolites were mapped to ‘Human Metabolome Database’ (HMDB) identifiers and linked to metabolic pathway according to KEGG database. The pathway enrichment analysis coupled with pathway topology analysis was performed on the metabolites that could be mapped to KEGG to identify significantly enriched pathways. The enrichment analysis was based on a global test and the metabolite importance was measured by relative betweenness centrality. Obtained *P*-values from the enrichment analysis were adjusted by Holm method. Adjusted *P*-values lower than 0.05 were considered significant.

## Results

### Demographic and clinical characteristics

The cohort comprised 138 patients who were distributed across three groups; patients who turned out to have sepsis (*N* = 49), patients who turned out to have non-septic infection (*N* = 47), and symptomatic controls that were patients in the ambulance who turned out to be infection free (*N* = 42). The demographic and clinical characteristics of the patients with are provided in Table 1. The median age varies among the groups, patients with sepsis having a median age of 80 years (IQR: 73–87), patients with non-septic infection at 77 years (IQR: 68–83), and the symptomatic controls at 74 years (IQR: 63–82) and there was no statistically significant age difference between the three groups (*P*-value = 0.102). The sex distribution was similar among patients with sepsis and patients with non-septic infection, 40% and 36% respectively while 61% of the symptomatic controls were female (*P*-value = 0.037).

Ambulance measured vital parameters indicate differences in respiratory, cardiovascular, and body temperature measures (Table 1). There were significant differences in respiratory rates (*P*-value = 0.0001), oxygen saturation (*P*-value = 0.0001), heart rates (*P*-value = 0.0001) where increased values were reported in the patients who developed sepsis and non-septic infection as compared to the symptomatic controls. Systolic blood pressure was significantly lower in patients who developed sepsis and non-septic infection vs symptomatic controls (*P*-value = 0.0180). In terms of neurological status, the GCS score was found to reflect full alertness (15) across the groups. Body temperature measurements in the ambulance were found to significantly different where the patients who developed sepsis were found to show a higher median temperature of 38.5 °C (IQR: 37.4–39.1), followed by the patients who developed non-septic infection at 37.8 °C (IQR: 37.4–38.7), and the symptomatic controls with the lowest median temperature of 36.7 °C (IQR: 36.3–37.1).

Blood tests showed that there were no significant differences between glucose and lactate between the groups (*P*-value > 0.05) but that there were significant differences in concentrations of suPAR and P-HBP.

### Metabolomics and lipidomics profiles of patients who developed sepsis compared to symptomatic control

**Fig. 2:**
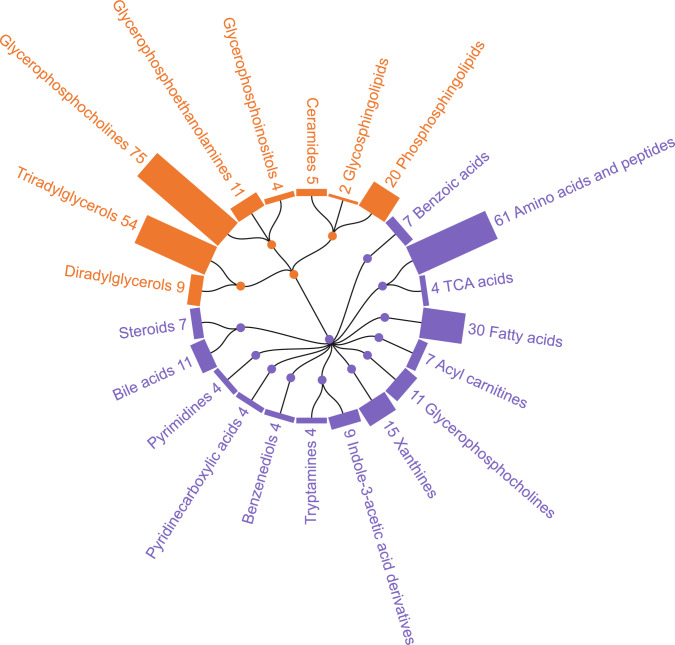
Coverage and class distribution of the plasma metabolites as measured by the metabolomics and lipidomics platforms in the present study. Pie chart with color indicating super-/sub-classes and size of segments representing the number of metabolites in the data set corresponding to the respective sub-class

A total of 457 metabolites, including metabolites acquired thorough the polar and semipolar UHPLC–MS/MS and UHPLC–QTOFMS platforms which were found to cover a broad range of metabolites from classes such as amino acids and peptides, fatty acids, xanthines, bile acids, glycerophosphocholines, organic acids and derivatives, acylcarnitines, steroids, pyrimidines, benzenediols, TCA acids, tryptamines, and pyridine carboxylic acids (Fig. 2). Correspondingly, the lipidomics platform covered molecular lipids from classes such as glycerophosphocholines, triacylglycerols, phosphosphingolipids, Glycerophosphoethanolamines, diacylglycerols, ceramides, glycerophosphoinositols, steroids, and glycosphingolipids (Fig. 2).

**Fig. 3:**
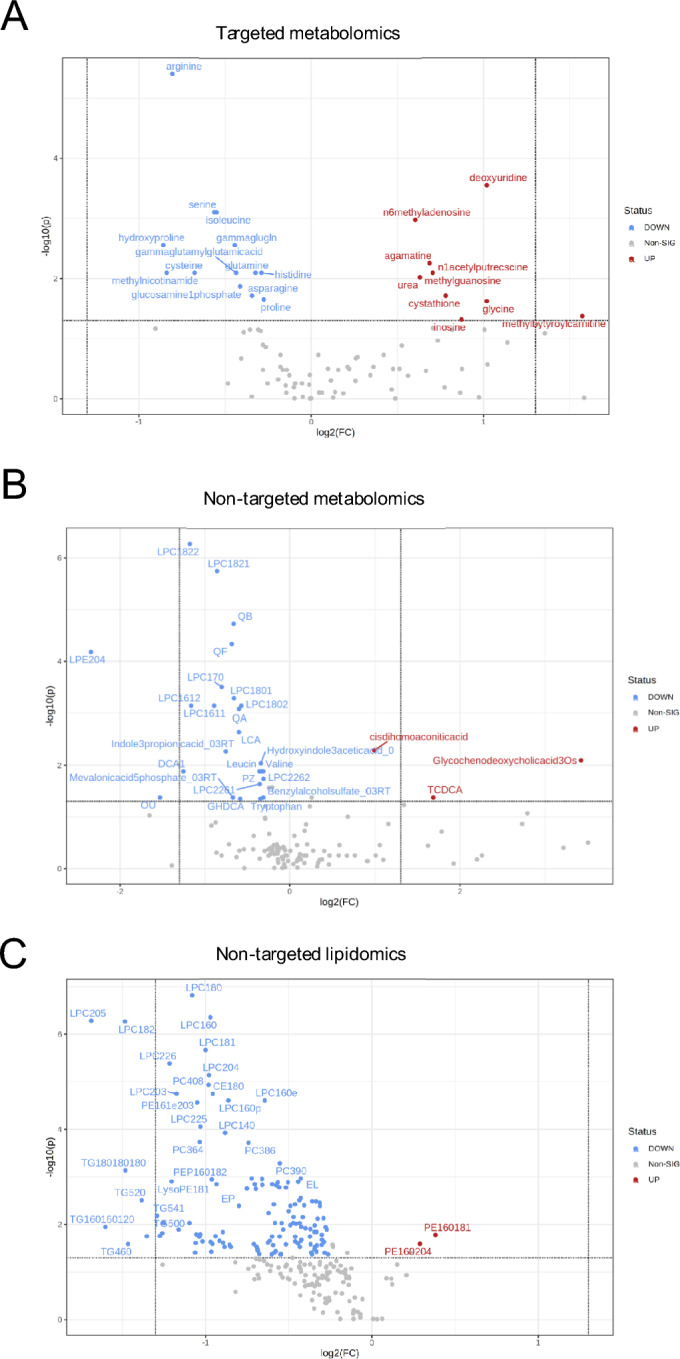
Univariable analysis identified significant differences (FDR-corrected *P*-value < 0.05) in metabolite levels in the comparison of sepsis vs symptomatic control. Metabolites found to be significantly decreased in sepsis are shown in blue, while significantly increased metabolites are highlighted. **A** 23 polar metabolites, mainly amino acids, were identified. Thirteen metabolites were significantly decreased, while ten metabolites, belonging to different classes (nucleosides, amino acids, and pyrimidines), were significantly increased. **B** 28 semipolar metabolites were identified. Twenty-three metabolites, mainly polar molecular lipids such as lysophosphatidylcholines, were significantly decreased, while three metabolites, including the bile acids glycochenodeoxycholic acid (GCDCA) and taurochenodeoxycholic acid (TCDCA), were significantly increased. **C** 133 molecular lipids were identified. The majority were significantly decreased

To identify distinctive metabolomic and lipidomic profiles indicative of early-stage sepsis in blood samples collected during ambulance transit, we first performed univariable analyses, comparing the three different metabolite and molecular platforms among patients who subsequently developed sepsis with symptomatic controls (Fig. 3). The univariable analysis revealed statistically significant differences (FDR-corrected *P*-value < 0.05) in the levels of 23 metabolites measured using targeted UHPLC–MS/MS metabolomics when comparing sepsis to symptomatic controls (Fig. 3A). Among these, 13 metabolites, predominantly amino acids, exhibited a significant decrease in septic conditions (blue), whereas ten metabolites including classes such as nucleosides, amino acids, and pyrimidines demonstrated a significant increase (red).

Turning to the semipolar platform, we observed statistically significant differences (FDR-corrected *P*-value < 0.05) in the metabolite levels of 28 semipolar metabolites when comparing patients who developed sepsis with symptomatic controls. Among these metabolites, 23, predominantly comprising molecular lipids such as lysophosphatidylcholines (LPC), demonstrated a significant decrease in the patients who developed sepsis (blue). Three metabolites, specifically the bile acids glycochenodeoxycholic acid (GCDCA) and taurochenodeoxycholic acid (TCDCA), were found to be increased in sepsis (Fig. 3B). Lastly, univariable analysis revealed significant differences (FDR-corrected *P*-value < 0.05) in the levels of 133 molecular lipids when comparing sepsis to symptomatic controls. Interestingly, most of these lipids exhibited a significant decrease in sepsis (blue), while only two molecular lipids, phosphoethanolamine (PE) 16:0/18:1 and PE (16:0/20:4), were found to be increased (Fig. 3C).

**Fig. 4:**
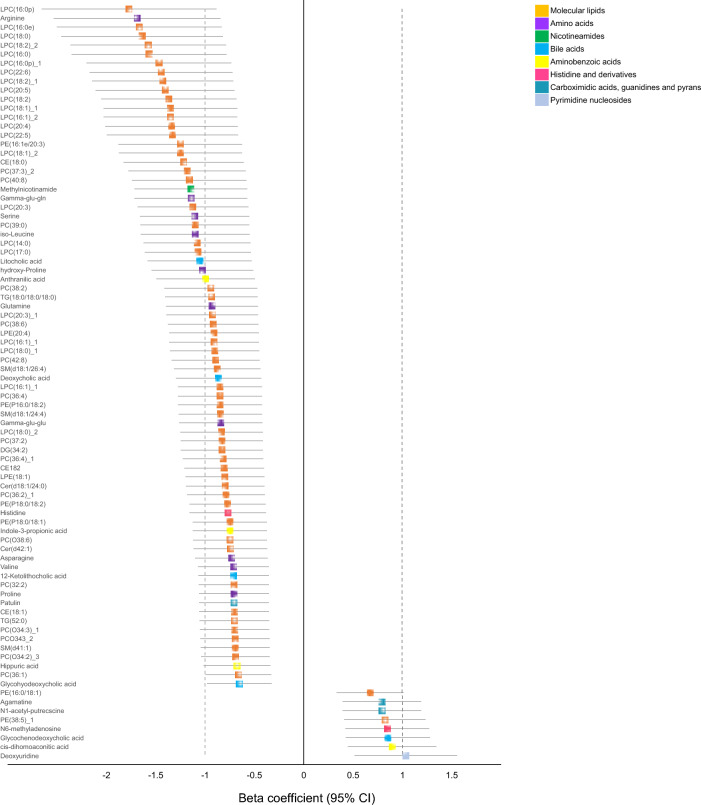
Multivariable logistic regression analysis identified 84 metabolites including molecular lipids that were significantly different in sepsis compared symptomatic control following adjustment for age, sex, and Charlson comorbidity score. Most of the metabolites and molecular lipids, 76 in total, were lower in sepsis, while 7 metabolites were found to be higher in sepsis vs symptomatic control. Isomers of molecular lipids are annotated with and asterisk (*)

Given the previously established association of age, sex, cardiometabolic disease, and other health conditions with metabolomics and lipidomic profiles (Auro et al., 2014; Ottosson et al., 2022; Rist et al., 2017), we performed a multivariable analysis while adjusting for age, sex, and the Charlson comorbidity score. Following multiple correction, we observed that 84 metabolites and molecular lipids in total were significantly different between the patients who developed sepsis compared to symptomatic control (Fig. 4). Out of the 84 metabolites, 76 were found to be decreased while 8 were increased. The amino acids arginine, serine, iso-leucine, and glutamine as well as the lysophosphatidylcholines (LPC) 16:0p and LPC 16:0 were the top metabolites that were lower among the patients who developed sepsis compared to symptomatic controls (Fig. 3). On the contrary, phosphoethanolamine (PE16:0/18:1), agmatine, N1-acetyl-putrescine, PE (38:5), N6-methyladenosine, glycochenodeoxycholic acid, cis-dihomoaconitic acid, and deoxyuridine were found to be significantly increased among the patients who developed sepsis.

**Fig. 5:**
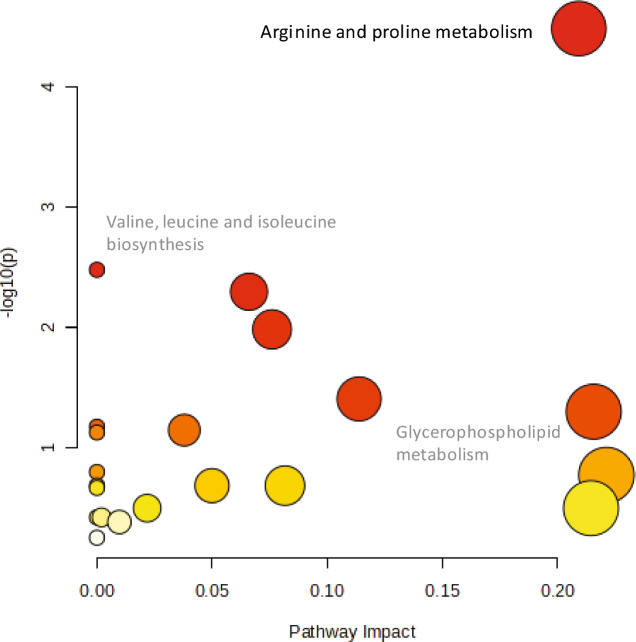
Pathway analysis, combining pathway enrichment and pathway topology analysis, of annotated metabolites and lipids in sepsis and symptomatic controls. The x-axis marks the pathway impact, while the y-axis represents the pathway enrichment. Each circle marks a pathway, with larger sizes and darker colors representing higher pathway impact values and higher pathway enrichment

Lastly, we performed a pathway analysis to identify the most relevant pathways involved in the present study. The top pathway that also displayed a significant enrichment was *Arginine and proline metabolism* (FDR corrected *P*-value = 0.003, Pathway impact = 0.209). Thereafter, *Valine, leucine and isoleucine biosynthesis* (FDR corrected *P*-value = 0.133, Pathway impact = 0.002), *Glycerophospholipid metabolism* (FDR corrected *P*-value = 0.662, Pathway impact = 0.216) (Fig. 5).

### Early metabolomics and lipidomics profiles did not separate patients with sepsis from patients with non-septic infection

We next sought to explore metabolomic and lipidomic profiles comparing patients with sepsis and patients with non-septic infection. No statistically significant differences (FDR-corrected *P*-value < 0.05) were observed in the levels of metabolites across the three platforms, polar metabolites, semipolar metabolites, and molecular lipids (Supplementary Fig. 1). Moreover, after adjusting for age, sex, and the Charlson comorbidity score, the results still indicated no significant differences in the metabolite and molecular lipid profiles between patients with sepsis from patients with non-septic infection. These findings suggest that the early metabolomics and lipidomics signatures in patients with sepsis do not distinctly separate from patients with non-septic infection.

### Relationship between key metabolites and clinical features in sepsis vs symptomatic controls

**Fig. 6:**
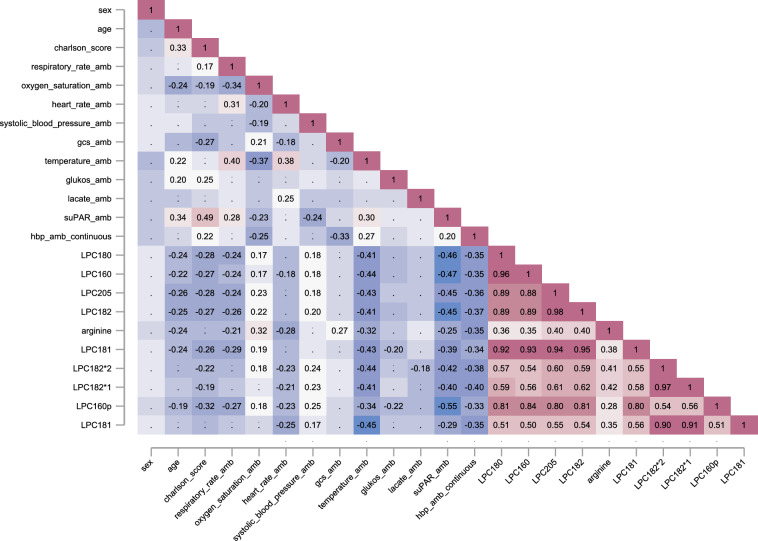
Pairwise correlations between age, sex, vital signs, blood tests, and comorbidity scores and the top ten metabolites. Only significant and FDR-corrected (*P*-value < 0.05) correlations are depicted. Isomers of molecular lipids are annotated with and asterisk (*)

Having identified 84 metabolites, with the majority showing decreased levels in sepsis, we next investigated the relationship between the most significant metabolites and clinical features. Specifically, we investigated the pairwise correlations between the top ten most significant metabolites with age, sex, vital parameters, and blood test results (Fig. 6). We identified significant (*P* < 0.05) negative correlations between several of the top amino acid arginine and lipids with clinical parameters such as Charlson comorbidity score, ambulance measured body temperature, levels of su-PAR and HBP.

## Discussion

This study describes the metabolomics and lipidomics profiles during the early stages of sepsis in blood samples collected during an ambulance transit. The main findings of our study are the following: first, we identified an overall decrease in the majority of significant circulating metabolites as a characteristic profile of patients who developed sepsis within 36 h from emergency department arrival. Second, we identified significant differences in amino acids and molecular lipids including primarly lysophosphatidylcholine and phosphatidylcholine between patients who develop sepsis compared to symptomatic control following adjustment for important covariates such as age, sex, and the Charlson comorbidity score. Additionally, bile acids, nucleosides, and small organic acids were found to differ between the two groups. At metabolic pathway level, we observed that amino acid metabolism and glycerophospholipid biosynthesis were important in sepsis. Lastly, we show that the early circulating metabolites including molecular lipids do not separate patients with sepsis from patients with non-septic infection.

### Amino acids

In the present study, we identified amino acid metabolism as a biochemical pathway of importance in sepsis and with most of the detected amino acids (arginine, proline, l-gamma-glutamyl-l-glutamine, iso-leucine, serine, hydroxyproline, glutamine, histidine, valine, asparagine) being significantly decreased among patients in the ambulance who developed sepsis compared to symptomatic controls.

Amino acid metabolism is an important contributor to metabolic pathways that support immunity, including ATP generation, nucleotide synthesis, and redox balance (Kelly & Pearce, 2020). Immune cells use these pathways to acquire energy and biomass, adapting their metabolism to facilitate diverse immune cell functions beyond simply enhancing protein synthesis (Kelly & Pearce, 2020).

There are inconsistencies in the literature regarding the levels of amino acids in septic patients, with some studies reporting higher levels (Lin et al., 2020; Mierzchała-Pasierb et al., 2021) and others reporting lower levels (Kauppi et al., 2016; Mickiewicz et al., 2014; Reisinger et al., 2021) compared to healthy controls. These inconsistent findings may be attributed to differences in study design, the metabolomics platforms employed (1H-NMR, GC–MS, and LC–MS), as well as small study sizes, which collectively introduce challenges in the generalization potential and robustness of each study.

Differences in amino acid levels and alterations of amino acid pathways have been identified in bacterial infections (Chen et al., 2022). In a study involving 53 adult medical ICU sepsis patients and 25 ICU controls without sepsis, proton nuclear magnetic resonance (1H-NMR) spectroscopy metabolomic analysis reported that decreased branched chain amino acid (BCAA) levels were associated with ICU and 28-day mortality (Reisinger et al., 2021). In another metabolomics study including a total of 63 patients with sepsis and 43 normal controls, differences in metabolic pathways that included phenylalanine, tyrosine and tryptophan biosynthesis, and arginine biosynthesis were reported between the groups (Chen et al., 2022). In a combined microbiome and metabolome investigation of a small sepsis cohort, a reduction in amino acid metabolism, including tryptophan, tyrosine, and arginine and proline metabolism, were reported (Sun et al., 2023). Moreover, the study reported that these metabolites were associated with an altered composition of bacteria as well as with more severe clinical outcomes, overall suggesting that these amino acids may play a role in the length of hospital stay among critically ill patients (Sun et al., 2023).

In the present study, arginine showed the highest reduction among patients who developed sepsis compared to symptomatic controls. Arginine, which serves as an intermediary in the urea cycle, also participates in the production of proteins, polyamines, creatine, and nitric oxide. In mild inflammation, the equilibrium between arginine production and breakdown tends to stay stable. However, during severe inflammation, arginine breakdown surpasses its biosynthesis in response to the increased utilization via the arginase and nitric oxide pathways (Luiking et al., 2005; Mickiewicz et al., 2014). As a result, decreased plasma arginine levels are anticipated and have been reported both in this study and in prior research (Mickiewicz et al., 2015; Mierzchała-Pasierb et al., 2021; Rath et al., 2014; Su et al., 2015). Finally, arginine serves as a precursor for the synthesis of agmatine, which we found to be significantly increased in the present study. The effects of arginine in severe inflammation has been investigated in two intervention studies, one of short duration (8 h) in patients with sepsis (Luiking et al., 2005) and the other for a longer duration (72 h) and among critically ill patients (Luiking et al., 2020). The short-term study reported that intravenous arginine infusion in sepsis increased plasma arginine levels, stimulating the production of new arginine and nitric oxide, and decreasing overall protein breakdown (Luiking et al., 2005). Conversely, the long-term study concluded that prolonged intravenous administration of arginine did not enhance local perfusion or organ function, despite an increase in whole-body nitric oxide synthesis (Luiking et al., 2020).

### Phospholipids

The present study also showed a marked reduction in the majority of phospholipids in patients who developed sepsis compared to symptomatic controls. This finding is largely in line with multiple previous studies where decreases in circulating lipoproteins, cholesterol, and molecular lipids including fatty acids, lysophosphatidylcholines, phosphatidylcholines, and sphingomyelins have been reported in sepsis and its associated severity and mortality (Cho et al., 2012; Drobnik et al., 2003; Kauppi et al., 2016; Mosevoll et al., 2023; Park et al., 2019). The disruption of cell signaling pathways, insufficient resolution of inflammation, and an imbalance in energy regulation, all of which undergo alterations in the context of sepsis, have been suggested to be factors that may contribute to the observed changes of lipid metabolism in sepsis (Amunugama et al., 2021).

We primarly observed a significand reduction of several LPCs in patients who developed sepsis as compared to symptomatic controls. This finding is corroborated by a study of plasma LPC in patients admitted to the intensive care unit with severe sepsis or septic shock (Park et al., 2014). The study also found that survivors show a gradual increase in serum and plasma LPC levels over time, whereas non-survivors did not (Park et al., 2014). Similar results of decreased LPC in sepsis have been reported previously (Drobnik et al., 2003; Ferrario et al., 2016).

The molecular mechanisms underlying the marked reduction of plasma LPC levels, precursors, metabolites, and relevant enzymes were investigated in an experimental model of sepsis using CLP-mice (Ahn et al., 2017). A decrease in plasma LPC was reported to be the result of an attenuated lecithin-cholesterol acyltransferase and secretory phospholipases A2 that was accompanied with an increase in LPCAT1-3 activity, suggesting that sepsis triggers LPC conversion to PC (Ahn et al., 2017). Similar effects were also observed in a study of the therapeutic effects of LPC in experimental sepsis (Yan et al., 2004). Increased PC and acylcarnitines have been observed in sepsis compared to SIRS (Schmerler et al., 2012). Taken together, these findings suggest that during systemic inflammation, the decrease in LPCs and increase in PCs represent a distinct host response to infection-induced inflammation.

In our study, there were no identified metabolites significantly differentiating between sepsis and non-septic infection. This remains challenging and maybe due to their common mechanistic pathways, in addition to heterogenous clinical presentations, i.e. a reflection of sepsis being a syndrome rather than a specific entity. Previous studies show inconclusive results. Conventional biomarkers such as procalcitonin and C-reactive protein have been shown to lack sufficient discriminatory power (Parlato et al., 2018), while other studies support that metabolomics and lipidomics profiles can separate infection from non-infection (Neugebauer et al., 2016). In addition, Parlato et al. (2018) did report that no combination of 29 plasma metabolites, 10 whole blood RNAs, and 14 leukocyte surface markers outperformed CRP alone in discriminating sepsis from non-septic SIRS. Despite these conflicting results, we believe that metabolomics approaches should continue to be explored as it provides a metabolic snapshot that could improve early identification.

### Strengths and limitations

Although the study design with standardized sample collection already in the ambulance transit provides a unique opportunity to investigate and detect early metabolomics and lipidomics profiles of sepsis, it was not possible to standardize the sampling regarding food intake and circadian differences. Comparing sepsis patients with symptomatic controls, rather than entirely healthy individuals, reflects a more clinically relevant scenario, which we consider a strength of our study. Since the present study is of a small sample size, with all participants originating from one region, verification of our findings in independent cohorts is warranted.

## Conclusion

In conclusion, our study explored the early metabolomic and lipidomic profiles in sepsis, highlighting amino acids and LPCs as particularly different compared to symptomatic controls. Although we were unable to differentiate between sepsis and non-septic infection, larger-scale investigations may be required to uncover potentially subtle differences between these conditions. This could have implications for the diagnosis and management of patients with suspected infections or sepsis, potentially leading to improved clinical outcomes through more accurate and timely interventions.

## Supplementary Information

Below is the link to the electronic supplementary material.

Supplementary file1 (PDF 134 KB)

## Data Availability

The data supporting the findings of the study are not publicly accessible and will be made accessible upon reasonable requested to the corresponding author (Lisa Kurland).

## References

[CR1] Ahn, W.-G., Jung, J.-S., Kwon, H. Y., & Song, D.-K. (2017). Alteration of lysophosphatidylcholine-related metabolic parameters in the plasma of mice with experimental sepsis. *Inflammation,**40*, 537–545. 28028754 10.1007/s10753-016-0500-6

[CR2] Amunugama, K., Pike, D. P., & Ford, D. A. (2021). The lipid biology of sepsis. *Journal of Lipid Research,**62*, 100090. 34087197 10.1016/j.jlr.2021.100090 PMC8243525

[CR3] Auro, K., Joensuu, A., Fischer, K., Kettunen, J., Salo, P., Mattsson, H., Niironen, M., Kaprio, J., Eriksson, J. G., Lehtimäki, T., Raitakari, O., Jula, A., Tiitinen, A., Jauhiainen, M., Soininen, P., Kangas, A. J., Kähönen, M., Havulinna, A. S., Ala-Korpela, M., … Perola, M. (2014). A metabolic view on menopause and ageing. *Nature Communications,**5*(1), 4708. 25144627 10.1038/ncomms5708

[CR4] Charlson, M., Szatrowski, T. P., Peterson, J., & Gold, J. (1994). Validation of a combined comorbidity index. *Journal of Clinical Epidemiology,**47*(11), 1245–1251. 7722560 10.1016/0895-4356(94)90129-5

[CR5] Chen, Q., Liang, X., Wu, T., Jiang, J., Jiang, Y., Zhang, S., Ruan, Y., Zhang, H., Zhang, C., Chen, P., Lv, Y., Xin, J., Shi, D., Chen, X., Li, J., & Xu, Y. (2022). Integrative analysis of metabolomics and proteomics reveals amino acid metabolism disorder in sepsis. *Journal of Translational Medicine,**20*(1), 123. 35287674 10.1186/s12967-022-03320-y PMC8919526

[CR6] Cho, W. H., Park, T., Park, Y. Y., Huh, J. W., Lim, C. M., Koh, Y., Song, D. K., & Hong, S. B. (2012). Clinical significance of enzymatic lysophosphatidylcholine (LPC) assay data in patients with sepsis. *European Journal of Clinical Microbiology & Infectious Diseases,**31*(8), 1805–1810. 22167258 10.1007/s10096-011-1505-6

[CR7] Drobnik, W., Liebisch, G., Audebert, F.-X., Fröhlich, D., Glück, T., Vogel, P., Rothe, G., & Schmitz, G. (2003). Plasma ceramide and lysophosphatidylcholine inversely correlate with mortality in sepsis patients. *Journal of Lipid Research,**44*(4), 754–761. 12562829 10.1194/jlr.M200401-JLR200

[CR8] Ferrario, M., Cambiaghi, A., Brunelli, L., Giordano, S., Caironi, P., Guatteri, L., Raimondi, F., Gattinoni, L., Latini, R., & Masson, S. (2016). Mortality prediction in patients with severe septic shock: A pilot study using a target metabolomics approach. *Scientific Reports,**6*(1), 20391. 26847922 10.1038/srep20391 PMC4742912

[CR9] Kauppi, A. M., Edin, A., Ziegler, I., Mölling, P., Sjöstedt, A., Gylfe, Å., Strålin, K., & Johansson, A. (2016). Metabolites in blood for prediction of bacteremic sepsis in the emergency room. *PLoS ONE,**11*(1), e0147670. 26800189 10.1371/journal.pone.0147670 PMC4723089

[CR10] Kelly, B., & Pearce, E. L. (2020). Amino assets: How amino acids support immunity. *Cell Metabolism,**32*(2), 154–175. 32649859 10.1016/j.cmet.2020.06.010

[CR11] Lin, S. H., Fan, J., Zhu, J., Zhao, Y. S., Wang, C. J., Zhang, M., & Xu, F. (2020). Exploring plasma metabolomic changes in sepsis: A clinical matching study based on gas chromatography-mass spectrometry. *Annals of Translational Medicine,**8*(23), 1568. 33437767 10.21037/atm-20-3562 PMC7791264

[CR12] Linder, A., Arnold, R., Boyd, J. H., Zindovic, M., Zindovic, I., Lange, A., Paulsson, M., Nyberg, P., Russell, J. A., Pritchard, D., Christensson, B., & Åkesson, P. (2015). Heparin-binding protein measurement improves the prediction of severe infection with organ dysfunction in the emergency department. *Critical Care Medicine,**43*(11), 2378–2386. 26468696 10.1097/CCM.0000000000001265 PMC4603368

[CR13] Luiking, Y. C., Poeze, M., & Deutz, N. E. (2020). A randomized-controlled trial of arginine infusion in severe sepsis on microcirculation and metabolism. *Clinical Nutrition,**39*(6), 1764–1773. 31522785 10.1016/j.clnu.2019.08.013

[CR14] Luiking, Y. C., Poeze, M., Ramsay, G., & Deutz, N. E. P. (2005). The role of arginine in infection and sepsis. *Journal of Parenteral and Enteral Nutrition,**29*(1S), S70–S74. 15709548 10.1177/01486071050290S1S70

[CR15] Mickiewicz, B., Duggan, G. E., Winston, B. W., Doig, C., Kubes, P., & Vogel, H. J. (2014). Metabolic profiling of serum samples by 1H nuclear magnetic resonance spectroscopy as a potential diagnostic approach for septic shock. *Critical Care Medicine,**42*(5), 1140–1149. 24368342 10.1097/CCM.0000000000000142

[CR16] Mickiewicz, B., Tam, P., Jenne, C. N., Leger, C., Wong, J., Winston, B. W., Doig, C., Kubes, P., Vogel, H. J., & Network, A. S. (2015). Integration of metabolic and inflammatory mediator profiles as a potential prognostic approach for septic shock in the intensive care unit. *Critical Care,**19*, 1–12. 25928796 10.1186/s13054-014-0729-0 PMC4340832

[CR17] Mierzchała-Pasierb, M., Lipińska-Gediga, M., Fleszar, M. G., Lewandowski, Ł, Serek, P., Płaczkowska, S., & Krzystek-Korpacka, M. (2021). An analysis of urine and serum amino acids in critically ill patients upon admission by means of targeted LC–MS/MS: A preliminary study. *Scientific Reports,**11*(1), 19977. 34620961 10.1038/s41598-021-99482-8 PMC8497565

[CR18] Mosevoll, K. A., Hansen, B. A., Gundersen, I. M., Reikvam, H., Bruserud, Ø., Bruserud, Ø., & Wendelbo, Ø. (2023). Patients with bacterial sepsis are heterogeneous with regard to their systemic lipidomic profiles. *Metabolites,**13*(1), 52. 10.3390/metabo13010052 PMC9864715 36676977

[CR19] Neugebauer, S., Giamarellos-Bourboulis, E. J., Pelekanou, A., Marioli, A., Baziaka, F., Tsangaris, I., Bauer, M., & Kiehntopf, M. (2016). Metabolite profiles in sepsis: Developing prognostic tools based on the type of infection. *Critical Care Medicine,**44*(9), 1649–1662. 27097292 10.1097/CCM.0000000000001740

[CR100] Nygren, H., Seppänen-Laakso, T., Castillo, S., Hyötyläinen, T., Orešič, M. (2011). Liquid chromatography-mass spectrometry (LC-MS)-based lipidomics for studies of body fluids and tissues. *Metabolic Profiling: Methods and Protocols, 708*, 247–257. 10.1007/978-1-61737-985-7_15 21207295

[CR20] Ottosson, F., Smith, E., Ericson, U., Brunkwall, L., Orho-Melander, M., Di Somma, S., Antonini, P., Nilsson, P. M., Fernandez, C., & Melander, O. (2022). Metabolome-defined obesity and the risk of future type 2 diabetes and mortality. *Diabetes Care,**45*(5), 1260–1267. 35287165 10.2337/dc21-2402 PMC9174969

[CR21] Pang, Z., Lu, Y., Zhou, G., Hui, F., Xu, L., Viau, C., Spigelman Aliya, F., MacDonald Patrick, E., Wishart David, S., Li, S., & Xia, J. (2024). MetaboAnalyst 6.0: Towards a unified platform for metabolomics data processing, analysis and interpretation. *Nucleic Acids Research,**52*, W398–W406. 38587201 10.1093/nar/gkae253 PMC11223798

[CR22] Park, D. W., Kwak, D. S., Park, Y. Y., Chang, Y., Huh, J. W., Lim, C.-M., Koh, Y., Song, D.-K., & Hong, S.-B. (2014). Impact of serial measurements of lysophosphatidylcholine on 28-day mortality prediction in patients admitted to the intensive care unit with severe sepsis or septic shock. *Journal of Critical Care,**29*(5), 882.e885-882.e811. 10.1016/j.jcrc.2014.05.003 24961965

[CR23] Park, J.-M., Noh, J.-Y., Kim, M.-J., Yun, T. G., Lee, S.-G., Chung, K. S., Lee, E. H., Shin, M. H., Ku, N. S., Yoon, S., Kang, M.-J., Park, M. S., & Pyun, J.-C. (2019). MALDI-TOF mass spectrometry based on parylene-matrix chip for the analysis of lysophosphatidylcholine in sepsis patient sera. *Analytical Chemistry,**91*(22), 14719–14727. 31621295 10.1021/acs.analchem.9b04019

[CR24] Parlato, M., Philippart, F., Rouquette, A., Moucadel, V., Puchois, V., Blein, S., Bedos, J. P., Diehl, J. L., Hamzaoui, O., Annane, D., Journois, D., Boutieb, M. B., Estève, L., Fitting, C., Treluyer, J.-M., Pachot, A., Adib-Conquy, M., Cavaillon, J.-M., Misset, B., & Captain Study Group. (2018). Circulating biomarkers may be unable to detect infection at the early phase of sepsis in ICU patients: The CAPTAIN prospective multicenter cohort study. *Intensive Care Medicine,**44*(7), 1061–1070. 29959455 10.1007/s00134-018-5228-3

[CR101] Pluskal, T., Castillo, S., Villar-Briones, A., Oresic, M. (2010). MZmine 2: modular framework for processing, visualizing, and analyzing mass spectrometry-based molecular profile data. *BMC Bioinformatics, 11*, 395. 20650010 10.1186/1471-2105-11-395 PMC2918584

[CR25] Rath, M., Müller, I., Kropf, P., Closs, E. I., & Munder, M. (2014). Metabolism via arginase or nitric oxide synthase: Two competing arginine pathways in macrophages. *Frontiers in Immunology,**5*, 532. 25386178 10.3389/fimmu.2014.00532 PMC4209874

[CR26] Reinhart, K., Daniels, R., Kissoon, N., Machado, F. R., Schachter, R. D., & Finfer, S. (2017). Recognizing sepsis as a global health priority—A WHO resolution. *New England Journal of Medicine,**377*(5), 414–417. 28658587 10.1056/NEJMp1707170

[CR27] Reisinger, A. C., Posch, F., Hackl, G., Marsche, G., Sourij, H., Bourgeois, B., Eller, K., Madl, T., & Eller, P. (2021). Branched-chain amino acids can predict mortality in ICU sepsis patients. *Nutrients,**13*(9), 3106. 34578983 10.3390/nu13093106 PMC8469152

[CR28] Rist, M. J., Roth, A., Frommherz, L., Weinert, C. H., Krüger, R., Merz, B., Bunzel, D., Mack, C., Egert, B., Bub, A., Görling, B., Tzvetkova, P., Luy, B., Hoffmann, I., Kulling, S. E., & Watzl, B. (2017). Metabolite patterns predicting sex and age in participants of the Karlsruhe Metabolomics and Nutrition (KarMeN) study. *PLoS ONE,**12*(8), e0183228. 28813537 10.1371/journal.pone.0183228 PMC5558977

[CR29] Robson, W., Nutbeam, T., & Daniels, R. (2009). Sepsis: A need for prehospital intervention? *Emergency Medicine Journal,**26*(7), 535–538. 19546282 10.1136/emj.2008.064469

[CR30] Rudd, K. E., Johnson, S. C., Agesa, K. M., Shackelford, K. A., Tsoi, D., Kievlan, D. R., Colombara, D. V., Ikuta, K. S., Kissoon, N., Finfer, S., Fleischmann-Struzek, C., Machado, F. R., Reinhart, K. K., Rowan, K., Seymour, C. W., Watson, R. S., West, T. E., Marinho, F., Hay, S. I., … Naghavi, M. (2020). Global, regional, and national sepsis incidence and mortality, 1990–2017: Analysis for the Global Burden of Disease Study. *The Lancet,**395*(10219), 200–211. 10.1016/S0140-6736(19)32989-7 PMC6970225 31954465

[CR31] Schmerler, D., Neugebauer, S., Ludewig, K., Bremer-Streck, S., Brunkhorst, F. M., & Kiehntopf, M. (2012). Targeted metabolomics for discrimination of systemic inflammatory disorders in critically ill patients. *Journal of Lipid Research,**53*(7), 1369–1375. 22581935 10.1194/jlr.P023309 PMC3371248

[CR32] Singer, M., Deutschman, C. S., Seymour, C. W., Shankar-Hari, M., Annane, D., Bauer, M., Bellomo, R., Bernard, G. R., Chiche, J.-D., Coopersmith, C. M., Hotchkiss, R. S., Levy, M. M., Marshall, J. C., Martin, G. S., Opal, S. M., Rubenfeld, G. D., van der Poll, T., Vincent, J.-L., & Angus, D. C. (2016). The third international consensus definitions for sepsis and septic shock (sepsis-3). *JAMA,**315*(8), 801–810. 26903338 10.1001/jama.2016.0287 PMC4968574

[CR33] Su, L., Li, H., Xie, A., Liu, D., Rao, W., Lan, L., Li, X., Li, F., Xiao, K., & Wang, H. (2015). Dynamic changes in amino acid concentration profiles in patients with sepsis. *PLoS ONE,**10*(4), e0121933. 25849571 10.1371/journal.pone.0121933 PMC4388841

[CR34] Sun, S., Wang, D., Dong, D., Xu, L., Xie, M., Wang, Y., Ni, T., Jiang, W., Zhu, X., Ning, N., Sun, Q., Zhao, S., Li, M., Chen, P., Yu, M., Li, J., Chen, E., Zhao, B., Peng, Y., & Mao, E. (2023). Altered intestinal microbiome and metabolome correspond to the clinical outcome of sepsis. *Critical Care,**27*(1), 127. 36978107 10.1186/s13054-023-04412-x PMC10044080

[CR35] Wallgren, U. M., Järnbert-Pettersson, H., Sjölin, J., & Kurland, L. (2022). Association between variables measured in the ambulance and in-hospital mortality among adult patients with and without infection: A prospective cohort study. *BMC Emergency Medicine,**22*(1), 185. 36418966 10.1186/s12873-022-00746-x PMC9686088

[CR36] Wallgren, U. M., Sjölin, J., Järnbert-Pettersson, H., & Kurland, L. (2020). The predictive value of variables measurable in the ambulance and the development of the predict sepsis screening tools: A prospective cohort study. *Scandinavian Journal of Trauma, Resuscitation and Emergency Medicine,**28*, 1–14. 32586337 10.1186/s13049-020-00745-6 PMC7318751

[CR37] Wishart, D. S. (2019). Metabolomics for investigating physiological and pathophysiological processes. *Physiological Reviews,**99*(4), 1819–1875. 31434538 10.1152/physrev.00035.2018

[CR38] Yan, J.-J., Jung, J.-S., Lee, J.-E., Lee, J., Huh, S.-O., Kim, H.-S., Jung, K. C., Cho, J.-Y., Nam, J.-S., Suh, H.-W., Kim, Y.-H., & Song, D.-K. (2004). Therapeutic effects of lysophosphatidylcholine in experimental sepsis. *Nature Medicine,**10*(2), 161–167. 14716308 10.1038/nm989

