# Supplementary material for: Exploring the circulating metabolome of sepsis: metabolomic and lipidomic profiles sampled in the ambulance
Source: Metabolomics. 2024 Oct 5;20(5):111. doi: 10.1007/s11306-024-02172-5 (PMC11455889; doi:10.1007/s11306-024-02172-5)
Supplement: Supplementary file 1 — Supplementary file1 (PDF 134 KB) [file 11306_2024_2172_MOESM1_ESM.pdf]

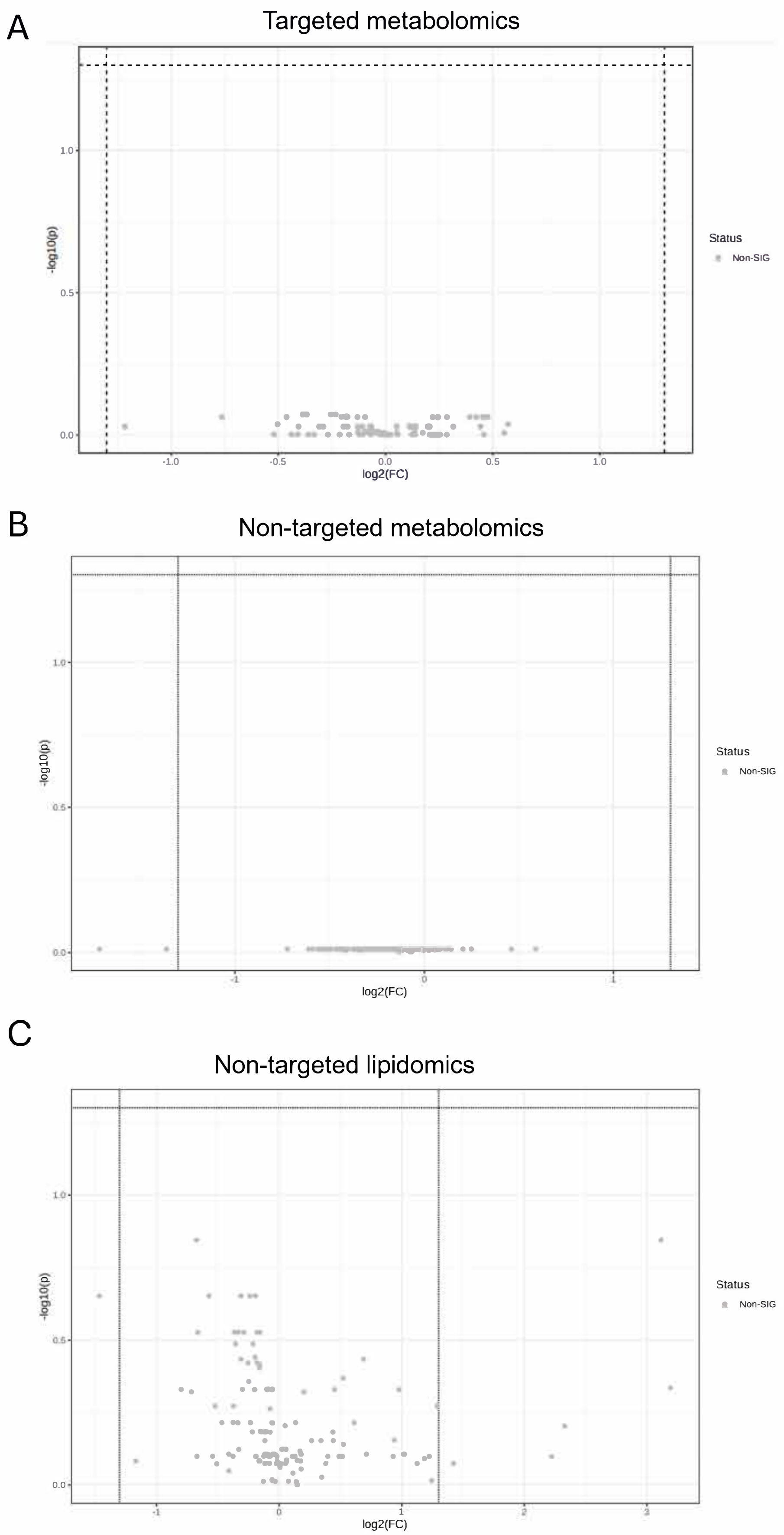

Supplementary Figure 1. Univariable analysis identified no significant differences (FDR-corrected P-value < 0.05) in metabolite levels in the comparison of sepsis vs non-septic infection across the three different platforms (A) Targeted metabolomics, (B) Non-targeted metabolomics, (C) Non-targeted lipidomics.
